# Autoantibodies in COVID-19 survivors with post-COVID symptoms: a systematic review

**DOI:** 10.3389/fimmu.2024.1428645

**Published:** 2024-07-05

**Authors:** Kin Israel Notarte, Timothy Hudson David Culasino Carandang, Jacqueline Veronica Velasco, Adriel Pastrana, Abbygail Therese Ver, Gerald Neil Manalo, Jeremy Ace Ng, Steven Grecia, Giuseppe Lippi, Brandon Michael Henry, César Fernández-de-las-Peñas

**Affiliations:** ^1^ Department of Pathology, Johns Hopkins University School of Medicine, Baltimore, MD, United States; ^2^ Pamantasan ng Lungsod ng Maynila College of Medicine, Manila, Philippines; ^3^ Department of Internal Medicine, Cardinal Santos Medical Center, San Juan, Philippines; ^4^ Faculty of Medicine and Surgery, University of Santo Tomas, Manila, Philippines; ^5^ College of Medicine, University of the Philippines Manila, Manila, Philippines; ^6^ Section of Clinical Biochemistry, University of Verona, Verona, Italy; ^7^ Clinical Laboratory, Division of Nephrology and Hypertension, Cincinnati Children’s Hospital Medical Center, Madrid, OH, United States; ^8^ Department of Physical Therapy, Occupational Therapy, Physical Medicine and Rehabilitation, Universidad Rey Juan Carlos, Madrid, Spain

**Keywords:** long COVID, PASC, post-COVID-19, autoantibodies, SARS-CoV-2 infection (COVID-19)

## Abstract

**Objective:**

The long-lasting persistence of autoantibodies stands as one of the hypotheses explaining the multisystemic manifestations seen in individuals with post-COVID-19 condition. The current review offers restricted insights into the persistence of autoantibodies in plasma/serum in people with post-COVID symptoms.

**Methods:**

PubMed/MEDLINE, CINAHL, EMBASE, and Web of Science databases, as well as on medRxiv and bioRxiv preprint servers were searched up to January 5^th^, 2024. Papers investigating the presence of autoantibodies in plasma/serum samples in people with post-COVID symptoms were included. The Newcastle-Ottawa Scale (NOS) was used to assess methodological quality.

**Results:**

From 162 identified records, five articles met all inclusion criteria; four studies included infected controls with no post-COVID symptoms whereas all five studies included non-infected controls (410 COVID-19 survivors with post-COVID symptoms, 223 COVID-19 survivors with no post-COVID symptoms as controls and 266 non-infected healthy controls). Four studies concluded that the presence of autoantibodies had a potential (but small) role in post-COVID-19 condition whereas one study concluded that autoantibodies were not associated. Quality assessment showed all studies had high methodological quality.

**Conclusion:**

Although evidence suggests that persistent autoantibodies can be associated with post-COVID symptoms, the clinical relevance of their presence seems modest at this stage. Current results highlight further research to clarify the role of autoantibodies in the development of post-COVID symptoms, guiding the development of tailored diagnostic and treatment approaches to enhance patient outcomes.

**Systematic review registration:**

https://osf.io/vqz28.

## Introduction

The coronavirus disease 2019 (COVID-19) caused by the Severe Acute Respiratory Syndrome Coronavirus 2 (SARS-CoV-2) may persist to pose a significant global health burden for the presence of long-lasting symptoms. Although most out of more than 772 million confirmed cases reported worldwide have recovered (1), post-COVID-19 condition (2) emerged as a significant concern affecting several SARS-CoV-2 infected individuals (3), translating into as many as 77 million affected people. Post-COVID-19 condition is a heterogeneous, multisystemic/multiorgan condition, displaying a vast clinical spectrum of long-term symptoms persisting after recovering from an acute SARS-CoV-2 infection (4). The most frequently reported symptoms are fatigue, dyspnea, and cognitive dysfunction, a variety of other symptoms may develop including (but not limited to) palpitations, chest pain, shortness of breath with exertion, presyncope, fatigue, pedal edema, sleep disorders, gastrointestinal problems, or irregular menstruation (4). A recent meta-analysis has found persistence of post-COVID symptoms in up to 30% of patients two years after an acute SARS-COV-2 infection (5). To date, the pathophysiology underlying post-COVID symptoms remains unknown, and several hypotheses including viral persistence, latent virus reactivation, microbiome dysbiosis, endotheliosis, microclots, and autoimmunity have been proposed (5, 6).

The persistent nature of post-COVID symptoms has become a subject of concern in current discussions in the medical field. Among the multifaceted factors explaining its prolonged nature, emerging evidence suggests that long-lasting presence of autoantibodies may underpin the persistent symptomatology observed in these patients (7, 8). Autoantibodies, acknowledged for their role in dysregulated immune response directed at self-antigens, have been proposed as responsible for prolonged inflammation and multisystemic manifestations seen at a post-COVID-19 phase (9, 10). Understanding the role and presence of these autoantibodies becomes essential for the development of targeted diagnostic procedures and therapeutic interventions to further improve patient outcomes (11). Current literature has predominantly concentrated on the presence of some autoantibodies particularly on G-protein coupled receptor (GPCR), and renin-angiotensin system autoantibodies, in individuals with post-COVID-19 condition (12). A comprehensive review has examined the spectrum of autoantibodies present in COVID-19 convalescents including anti-interferon antibodies, autoantibodies targeting the components of the cardiovascular system (including anti-cardiolipin, anti-lipoprotein A-1, and β2-glycoprotein autoantibody), thyroid autoantibodies (e.g., anti-thyroglobulin and anti-thyroid peroxidase), rheumatic disease associated autoantibodies (such as cyclic citrullinated peptide autoantibody or Rheumatoid factor antibody), GPCR autoantibodies, and other (e.g., anti‐SUMO1‐DHX35, and anti-calprotectin). These autoantibodies hold the potential to contribute to certain symptoms observed in individuals with post-COVID-19 syndrome (13). To the best of our knowledge, no previous review has systematically examined the persistence of these autoantibodies in serum/plasma samples in people experiencing lingering post- COVID symptoms. This review aimed to identify, appraise, and synthesize current evidence on the presence of autoantibodies in patients with post-COVID-19 condition.

## Methods

A systematic review of studies investigating the long-lasting presence of autoantibodies in plasma/serum samples in people with post-COVID symptoms according to the 2020 Preferred Reporting Items for Systematic reviews and Meta-Analyses (PRISMA) statement was performed (14). The review was prospectively registered in the Open Science Framework database (https://osf.io/vqz28).

Meta-analysis was not done because of the heterogeneity in the types of autoantibodies and the lack of controls, which provide crucial information about baseline values, and therefore, the magnitude of effect of the presence of autoantibodies on the occurrence of post-COVID symptoms.

### Literature search

Electronic literature searches were conducted by two authors for published studies up to January 5^th^, 2024 on PubMed/MEDLINE, CINAHL, EMBASE, and Web of Science databases, as well as on medRxiv and bioRxiv preprint servers. Searches were conducted with assistance of an experienced health science librarian. We screened the reference list of those papers for identifying other studies. The combinations of search terms using Boolean operators on each database are outlined in **Table 1**.

**Table 1:** Database formulas during literature search.

PubMed Search Formula
1 “post-acute COVID-19 syndrome” [MeSH Terms] OR “long-COVID” [All Fields] OR “long-COVID symptoms” [All Fields] OR “long hauler” [All Fields] OR “post-COVID-19” [All Fields] OR “post-acute COVID-19 symptoms” [All Fields] OR “COVID-19 sequelae” [All Fields] 2 “autoantibodies” [MeSH Terms] OR “autoantibody” [All Fields] 3 #1 AND #2

Medline/CINAHL (via EBSCO) Search Formula
1 (post-acute COVID-19 syndrome) OR (long-COVID) OR (long-COVID symptoms) OR (long hauler) OR (post-COVID-19) OR (post-acute COVID-19 symptoms) OR (COVID-19 sequelae) 2 (autoantibodies) OR (autoantibody) 3 #1 AND #2

EMBASE Search Formula
(post-acute COVID-19 syndrome) OR (long-COVID) OR (long-COVID symptoms) OR (long hauler) OR (post-COVID-19) OR (post-acute COVID-19 symptoms) OR (COVID-19 sequelae) AND (autoantibodies) OR (autoantibody)

### Selection criteria

The inclusion and exclusion criteria were described according to the Population, Intervention, Comparison and Outcome (PICO) principle:

Population: Adults (>18 years) who were previously infected by SARS-CoV-2 and diagnosed with real-time reverse transcription-polymerase chain reaction (RT-PCR) assay or SARS-CoV-2 serological test.

Intervention: Not applicable

Comparison: Not applicable

Outcome: Articles investigating the presence of autoantibodies in plasma/serum in patients with post-COVID symptoms. Articles should collect any post-COVID symptoms, such as fatigue, dyspnea, pain, brain fog, memory loss, skin rashes, palpitations, cough, as well as the presence of autoantibodies.

### Screening process, study selection and data extraction

Cohorts and case-control studies where the presence of autoantibodies in plasma/serum samples in patients who had developed post-COVID symptoms after an acute SARS-CoV-2 infection were included. Research letters or correspondence were included if they present new data. Case studies/case series, editorials or opinion articles without new data were excluded. Only studies including living individuals and full-text English language papers were considered. Post-mortem studies were excluded.

The title/abstract of each article identified during the database search was screened by two authors. First, duplicates were removed; full text of eligible articles was analyzed by the same authors. Data including authors, country, sample size, setting, age, post-COVID symptoms, sample of autoantibody analysis, and follow-up were extracted from each study. Both authors should have a consensus on study selection and data extraction. Discrepancies at any stage of the screening process were resolved by asking a third author if needed.

### Methodological quality/risk of bias

The Newcastle-Ottawa Scale (NOS), a nine-star rating system evaluating the risk of bias and methodological quality of observational (case-control and cohort) studies was applied by two authors (15). In cohort studies, the NOS evaluates the following items: case selection (i.e., cohort representativeness, selection of non-exposed cohort, case definition, outcome), comparability (i.e., proper control for age, sex, or other factors, between-group comparisons) and exposure (i.e., outcome assessment, enough and adequate follow-up). In case-control studies, NOS items are adapted. For instance, case selection item includes adequate case definition and control selection. Thus, the quality of longitudinal cohort studies or case-control studies is classified as: high quality (7–9 stars), moderate quality (5–6 starts), or low quality (≤4 stars). Methodological quality was also evaluated by two authors. If disagreement between both authors was identified, a third researcher arbitrated the final decision.

## Results

### Study selection

**Figure 1:**
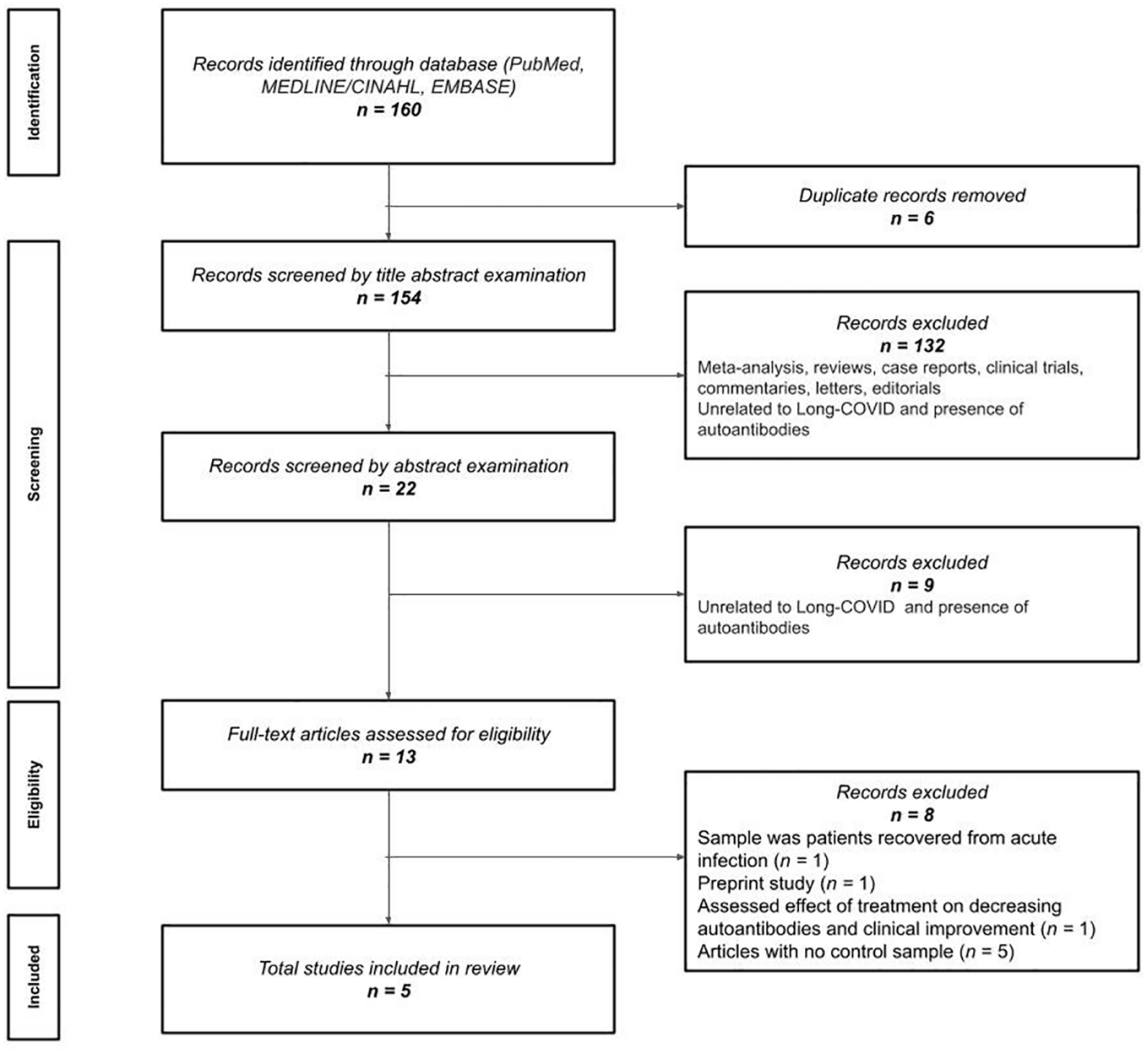
Preferred Reporting Items for Systematic reviews and Meta-Analyses (PRISMA) flow diagram.

The electronic literature search identified 162 records for screening. Duplicates were removed (n=6) and 156 records remained for title and abstract examination. One hundred thirty-two (n=132) were excluded in the initial title examination because they were meta-analysis, reviews, case reports, clinical trials, commentaries or editorials and mostly unrelated to post-COVID and the presence of autoantibodies. Nine (n=9) were excluded during their abstract examination because they did not assess autoantibodies in patients with post-COVID-19 condition. This led to thirteen articles assessed for full-text review, where two were excluded because the sample in the studies included COVID-19 survivors without mention to post-COVID symptomatology (16, 17), and one was excluded because it investigated changes in autoantibody levels after treatment (18), and five were excluded due to the absence of control samples (7, 19–22). Thus, a total of five (n=5) peer-reviewed studies (8–10, 23, 24) were finally included in this systematic review (**Figure 1**).

### Sample characteristics

The total sample included in the studies consisted of 647 COVID-19 survivors (410 COVID-19 survivors with post-COVID symptoms and 223 COVID-19 survivors without post-COVID symptoms) and 266 non-infected healthy controls. Four studies included COVID-19 survivors without post-COVID symptoms (8–10, 24) and all five studies included non-infected healthy controls (8–10, 23, 24). The specific post-COVID symptoms investigated in each study is listed in **Table 2**. All studies analyzed the presence of autoantibodies in serum/plasma samples (8–10, 23, 24). One study investigated the presence of SARS-CoV-2 antibodies from 4 to 14 months after infection (24), one study at 8 and 10 months after infection (9), and two studies in a range from 3 to 12 months after infection (8, 10). One study included analysis for antibodies from the day 0 of infection until 11 months after (23). **Table 2** summarizes the main findings of the studies included in this systematic review.

**Table 2:** Main findings of the studies included in the review about the presence of SARS-CoV-2 autoantibodies.

Author Country	Study Design Sample Size	Study Timeline	Mean/Median (Range)	Post-COVID Symptoms	Sample Analyzed	Follow-up Period	Main Findings
Son et al. (8) Canada	Longitudinal case-control COVID-19 survivors (n=106) Post-COVID symptoms 3 months after (n=50) No Post-COVID symptoms (n=42) Healthy non-infected controls (n=22)	August 2020 to September 2021	Mean (Range) 57 (20–89) y	Fatigue, cough, shortness of breath	Serum	3, 6, and 12 months after infection	**IgG autoantibodies in patients 3 months after:** Post-COVID patients with at least one autoreactive IgG: 13/36, 36% Post-COVID patients with two or more autoantigens: 12/36, 33% (IgG autoantibodies detected: 21/102 screen antigens (21%) IgG autoantibodies against nuclear or extractable nuclear antigens (ANAs/ENAs) 9/21 detected (43%): SS-B/LA, PM/Scl-75, M2, KS, PL-12, PM/Scl-100, Ribosomal P, Sm, dsDNA IgG autoantibodies with no known role in autoimmune diseases 12/21 (57%): ACE2, MDA5, CD255, Thyroglobulin, Calprotectin, LC, IFNa, IL-17F, TPO, GAD65, Aldolase C, Collagen VI ** Changes in circulating levels of ANAs/ENAs** Patients with 2 or more ANAs/ENAs 3 months after: 84/106, 79% Patients with 2 or more ANAs/ENAs 6 months after: 76/98, 78% Patients with 2 or more ANAs/ENAs 12 months after: 34/58, 41% Detectable ANAs/ENAs by 12 months (prevalence rate for n=57 patients): anti-SmD1 (11%), anti-PCNA (9%), anti-SS-A/Ro60 (12%), anti-SS-B/La (21%), anti-U1-snRNP (30%), anti-PM-Scl (21%), anti-Ku (11%), anti-DFS70 (12%) Serum samples with positive ANA reactivities at 12 months but previously below cut-off threshold at 3 or 6 months: 10/85 (12%) ** ANAs/ENAs and symptoms in post-COVID patients**: ANA/ENA frequencies 3 months after: 54% ANA/ENA frequencies 6 months after: 77% ANA/ENA frequencies at 12 months: 50%
Seibert et al. (10) Germany	Cross-sectional case-control COVID-19 survivors (n=130) Post-COVID symptoms (n=72) No post-COVID symptoms (n=58) Healthy non-infected controls (n=70)	Data not available	Median (Range) Post-COVID symptoms 48 (24–71) y No Post-COVID symptoms 50 (18–86) y Controls 50 (18–82) y	ISARIC COVID-19 study protocol (https://isaric.org/research/covid-19-clinical-research-resources/covid-19-long-term-follow-up-study-old/). Neurological symptoms and fatigue	Serum	12 months after infection	** Beta-1 Adrenergic Receptor (ADRB1)** Post-COVID symptoms: n=20 (27.8%) No Post-COVID symptoms: n=7 (12.1%) Controls: n=5 (7.1%) ** Beta-2 Adrenergic Receptor (ADRB2)** Post-COVID symptoms: n=16 (22.2%) No Post-COVID symptoms: n=14 (24.1%) Controls: n=7 (10%) ** Angiotensin-II-Receptor-1 (AGTR2)** Post-COVID symptoms: n=35 (48.6%) No Post-COVID symptoms: n=12 (20.7%) Controls: n=7 (10%) ** Muscarinergic Choline Receptor 3 (CHRM3)** Post-COVID symptoms: n=17 (23.6%) No Post-COVID symptoms: n=8 (13.8%) Controls: n=3 (4.3%) ** Muscarinergic Choline Receptor 4 (CHRM4)** Post-COVID symptoms: n=35 (48.6%) No Post-COVID symptoms: n=7 (12.1%) Controls: n=9 (12.8%) ** Endothelin Receptor (EDNRA)** Post-COVID symptoms: n=7 (9.7%) No Post-COVID symptoms: n=7 (12.1%) Controls: n=5 (7.2%)
Schultheiß et al. (9) Germany	Cross-sectional cohort Discovery cohort (n=318) COVID-19 survivors (n=258) Post-COVID symptoms (n=175) No Post-COVID symptoms (n=83) Healthy non-infected controls (n=36)	Discovery cohort: until October 9, 2021 Validation cohort: until February 18, 2022	Median (Range) 51.3 (15–83) y	Fever, lymph node swelling, loss of taste/smell, body aches, fatigue, sleeping disturbances, lack of concentration, anxiety, depression, headache, coryza, conjunctivitis, dyspnea, sore throat, cough, heart complaints, dizziness, abdominal pain, gastrointestinal complications, nausea	Plasma	Discovery cohort: 8 months after infection Validation cohort: 10 months after infection	COVID-19 survivors: 20% positive for autoantibodies (RF, ANAs, aPLs) but not correlated with post-COVID symptoms ** Hallmark cytokine triad** of post-COVID symptoms: only IL-1b (p=0.01), IL-6 (p=0.04), and TNF (p<0.001) with correlation No time-dependent decrease of cytokine triad levels after resolution of symptoms
Acosta-Ampudia et al. (23) Colombia	Cross-sectional cohort Post-COVID symptoms (n=33) Healthy non-infected controls (n=100) from historical database	Data not available	Autoimmune assessment: Median: 55; Interquartile range: 50–63 Immunological assessment: Median: 50.5; Interquartile range: 49.75–55.5	Fatigue, headache, fever, dyspnea, dry cough, myalgia, arthralgia, ageusia, pharyngitis, anosmia, dizziness, diarrhea, rhinorrhea, impaired visual acuity, edema	Serum	D0, D4, D7, D14, D28, and 7 to 11 months after infection	**Autoantibody seroconversion from onset to post-COVID** ANA: slight increase at 1/80 dilution (*P* = .045) Other autoantibodies: no change (*P* >.05) ** Onset vs. PCS β2GP1 IgM autoantibodies:** increase (*P* = .0135) **Autoimmune assessment in acute phase vs. post-COVID** At least 1 autoantibody: 57.6% vs 63.6% (*P* = .0006) 2 or more autoantibodies: 33.3% versus 48.5% (*P* = .0007) ** Immunological assessment in acute phase vs. post-COVID** IgA anti-SARS-CoV-2 S1 antibodies: 22.3% reduction (*P* = .0257) IgG anti-SARS-CoV-2 S1 antibodies: 33.6% reduction (*P* <.0001) Note: IgA antibodies returned to similar levels from the acute phase, but IgG antibodies remained high (*P* <.0001)
Sotzny et al. (24) Germany	Cross-sectional cohort COVID-19 survivors (n=120) Post-COVID symptoms (n=80) No Post-COVID symptoms controls (n=40) Healthy non-infected controls (n=38)	August 2020 to July 2021	Median (Range) PCS/ME/CFS: 46.5 (24–62) y PCS/non-ME/CFS: 40 (22–67) y PCHC: 35 (21–66) y HC: 38 (19–64) y	Severe fatigue, intolerance to mental and physical exertion, cognitive impairment, muscle pain, headache, dry eyes, dry mouth, Raynaud symptoms	Serum	PCS/ME/CFS: 4–14 months after COVID infection PCS/non-ME/CFS: 4–13 months after COVID infection PCHC: 4–10 months after COVID infection	**Machine learning-supervised algorithms revealed stratification overlap between PCS, PCHC, and HC autoantibodies** ADRA2A, ADRB2, and STAB1 ** Lower concentrations of 10 out of 20 analyzed circulation autoantibodies in PCS vs. healthy control** **groups (PCHC and HC)** ADRA2A, ADRB2, BDKRB1, MAS1, CHRM5, CHRNA1, EDNRA, F2R/PAR-1, STAB1, CXCR3

HC, healthy control; ME/CFS, Myalgic Encephalomyelitis/Chronic Fatigue Syndrome; mo, months; PCHC, post-COVID-19 healthy control; PCS, post COVID Syndrome; y, years.

### Methodological quality

One cross-sectional case-control study, one longitudinal case- control study, and three cross-sectional cohort studies underwent methodological quality assessment (**Table 3**). No disagreement between both authors in methodological quality was observed. All five studies were of high methodological quality (mean: 8 SD: 0.7 stars) (8–10, 23, 24) (**Table 3**).

**Table 3:** Methodological quality (Newcastle-Ottawa Scale - NOS) of the studies included in the review.

Study	Selection				Comparability		Outcome			
	Representative of the exposed cohort	Selection of nonexposed cohort	Ascertainment of exposure	Outcome of interest	Main Factor	Additional factor	Assessment of outcomes	Sufficient follow-up	Adequacy of follow-up	Total Score
Son et al. (8)	★	★	★	★	★	★	★	★	★	9/9
Seibert et al. (10)	★	★	★	★	★		★		★	7/9
Schultheiß et al. (9)	★	★	★	★	★	★	★		★	8/9
Acosta-Ampudia et al. (23)	★	★	★	★		★	★	★	★	8/9
Sotzny et al. (24)	★	★	★	★	★		★	★	★	8/9

### Persistence of autoantibodies in plasma/serum

All studies reported the presence of autoantibodies in plasma/serum of patients previously infected by SARS-CoV-2, with varying prevalence rate and significance. While the majority of studies concluded that the presence of autoantibodies exhibited a potential, but small, role in post-COVID-19 condition (8, 10, 23, 24), one study had concluded that autoantibodies were not associated with this condition (9).

Among studies that correlated autoantibodies and development of post-COVID symptoms, Acosta-Ampudia et al. found the presence of at least one autoantibody among patients with post-COVID symptoms compared with those in the acute phase of illness (23). The case-control study conducted by Son et al. found that individuals with post-COVID symptoms had significantly higher levels of antinuclear antibodies (ANA) 3 months after acute infection (8). Thus, the severity of acute infection was also found to be correlated with a stronger autoimmune response, but the number of ANA autoantibodies decreased from 3 to 12 months after (8). Son et al. also saw that the presence of autoantibodies anti-U1-snRNP and anti-SS-B/La predicted the presence of post-COVID fatigue (specificity: 92%, sensitivity: 70%) and dyspnea (specificity: 97%, sensitivity: 58%) (8). In addition, Seibert et al. found that the incidence of patients positive for several autoantibodies was higher in patients with post-COVID symptoms, followed by those with SARS-CoV-2 infection without developing post-COVID symptoms, and was the lowest in healthy non-infected controls (10). The most frequent combination of autoantibodies in the post-COVID group was a-ADRB1: a-AGTR2: a-CHRM3: a-CHRM4 (10). This study also found that levels of autoantibody CHRM3 were associated with post-COVID fatigue, while no such association was found in the remaining autoantibodies (10). Sotzny et al., using machine learning-supervised algorithms found that autoantibodies against autonomic nervous system-related receptors ADRA2A and ADRB2, along with scavenger receptors STAB1, emerged as primary classifiers of post-COVID syndrome (24). This study also revealed significantly lower concentrations of ten out of the 20 analyzed circulating autoantibodies in post COVID syndrome groups compared to healthy control counterparts. Among the autoantibodies exhibiting reduction in post-COVID patients, those involved in regulating vascular tone (e.g., ADRA2A, ADRB2, BDKRB1, MAS1, CHRM5, CHRNA1, EDNRA, F2R/PAR-1) along with STAB1 which functions as a scavenger receptor and regulates angiogenesis, as well as the inflammatory chemokine receptor CXCR3, were prominent (24).

On the other hand, the study of. Schultheiß et al. determined that although 20% of COVID-19 survivors tested positive for at least one or more autoantibodies, these were not correlated with post-COVID-19 condition (9).

## Discussion

The emergence of long-lasting post-COVID symptomatology represents a significant challenge in current healthcare settings. This systematic review offers restricted insights into the persistence of autoantibodies in people experiencing post-COVID symptoms, to understand the underlying mechanisms beneath this condition. The findings of this review further support the heterogeneous nature of post-COVID-19 condition (often referred to as long-COVID), the different factors that contribute to its pathogenesis, and the different treatment protocols implemented depending on its phenotype. Quantitative syntheses in most of the studies demonstrate that patients with post-COVID symptoms exhibit higher autoantibody positivity rate than healthy controls, suggesting that long persistence of autoantibodies seems to be unique in patients with this condition. A long-lasting persistence of autoantibodies, known for their role in dysregulated immune responses targeting self-antigens, stands as one of the hypotheses explaining the multisystemic manifestations seen in post-COVID-19 condition (5). The majority of the studies included in this review reported the presence of autoantibodies in serum/plasma samples of individuals with known previous SARS-CoV-2 infection, suggesting its potential role in post-COVID symptomatology, although the clinical relevance seems to be small.

The persistence of some autoantibodies, e.g., anti-U1-snRNP, anti-SS-B/La, and CHRM3 autoantibody, in plasma (general sample) was associated with specific post-COVID symptoms such as fatigue and dyspnea (8, 10). However, a study concluded that the proportion of patients exhibiting post-COVID symptomatology and testing positive for autoantibodies was not significant to conclude a correlation (9). It should be noted that inconsistencies in the current literature regarding the association between long-lasting autoantibodies and post-COVID-19 conditions were observed. These discrepancies in the findings may be associated with differences in population, methodology, target autoantibodies, or extraction sample. Moreover, the complexity and wide spectrum of post-COVID symptoms itself may have contributed to the conflicting results observed across identified studies. Further research investigating immunological mechanisms underlying specific post-COVID symptoms is warranted, including the potential role of autoantibodies in neurologic sequelae.

Although this study is the first to summarize current evidence on the presence of autoantibodies and post-COVID-19 conditions, the results should be considered according to some limitations. First, due to the limited number of published studies, different study designs, heterogeneous methodologies, and inconsistent results, any definitive conclusion should be done with caution at this stage, and the generalizability of current findings cannot be substantiated. Lack of statistical power due to small sample size limited elucidation of the source of difference in autoantibody rates between post-COVID-19 condition affected and healthy patients, as reflected in the contrast between analyses of overall autoantibody rates and positivity rates for 1 and ≥2 autoantibodies. Second, we only included studies investigating the association of autoantibodies and post-COVID-19 conditions. Preliminary evidence suggests that treatments that are able to decrease the autoantibody levels are associated with clinical improvement of post-COVID symptoms (18). In a quasi-experimental study, therapeutic apheresis was found to decrease autoantibodies against ß1- and ß2-adrenergic receptors by 33% and 28%, respectively, and autoantibodies against M3- and M4-acetylcholine receptors were reduced by 48% and 39%, respectively. Thus, patients showing a significant reduction in these autoantibodies, lipids, and inflammatory markers reported significant improvement following two cycles of therapeutic apheresis in their post-COVID symptomatology (18). However, the absence of a control group that did not receive treatment prevents us from concluding that the decrease in autoantibody levels is not associated with the passage of time, since the result of our review supports that levels of autoantibodies are lower with longer follow-up periods. Third, all studies included in the current review did not obtain a baseline autoantibody level prior to SARS-CoV-2 infection. As such, the studies did not account for patients who may have had an undocumented underlying autoimmune disease. This confounding factor could have skewed the results as patients with *de novo* autoantibodies may have more symptoms of long-COVID after acute infection owing to their already elevated autoantibodies even prior to infection. Fourth, we only included studies investigating adults, and thus cannot apply our results to children. Our literature search conducted up to January 5th 2024, did not reveal any study regarding autoantibodies and post-COVID in children. The heterogeneity in the duration of patient follow-up and the lack of data regarding the severity of acute infection within the post-COVID syndrome population across the current scientific literature is another source of uncertainty in our findings. Lastly, although the countries where the studies were performed and study timelines were included in **Table 2** when available, correlation between a specific COVID-19 variant and post-COVID symptoms cannot be made. Likewise, the number of exposures, repeated or multiple infections, and the possible enhancement of post-COVID symptoms and the presence of autoantibodies were not discussed in the included studies and therefore cannot be included in this review. Given the available evidence, future studies investigating the presence of autoantibodies and post-COVID-19 conditions over well-defined periods of time should take note of these gaps identified in this review.

## Conclusion

This systematic review provides an insight regarding the intricate relationship between persistent autoantibodies and post-COVID symptoms. Furthermore, this review presents strong evidence that patients with post-COVID symptoms have higher autoantibody positivity rate compared to healthy non-infected controls. While the majority of the evidence suggests a potential association between the presence of autoantibodies and post-COVID-19 conditions, the clinical significance appears to be small, and conflicting results were observed across the studies. The presence of autoantibodies, particularly in plasma/serum, may contribute to the ongoing inflammation and multisystemic manifestations found in patients with post-COVID symptoms. The limited number of published studies, variation in methodology, population differences, heterogeneity of post-COVID symptoms, and the lack of studies that obtained baseline autoantibody levels prior to COVID infection underscores the need for further research to identify the precise mechanisms that underlie this association. Further studies acknowledging these gaps would be essential for guiding tailored diagnostic and treatment approaches to enhance patient care and outcomes in the context of post-COVID-19 conditions.

## Data availability statement

The original contributions presented in the study are included in the article/supplementary material. Further inquiries can be directed to the corresponding authors.

## Author contributions

KN: Writing – review & editing, Writing – original draft, Visualization, Validation, Supervision, Software, Resources, Project administration, Methodology, Investigation, Funding acquisition, Formal analysis, Data curation, Conceptualization. TC: Writing – review & editing, Writing – original draft, Validation, Methodology, Data curation. JV: Writing – review & editing, Writing – original draft, Validation, Methodology, Formal analysis. AP: Writing – review & editing, Writing – original draft, Visualization, Methodology, Formal analysis, Data curation. AV: Writing – review & editing, Writing – original draft, Validation, Methodology, Formal analysis, Data curation. GM: Writing – review & editing, Writing – original draft, Validation, Methodology, Formal analysis, Data curation. JN: Writing – review & editing, Writing – original draft, Validation, Methodology, Formal analysis, Data curation. SG: Writing – review & editing, Writing – original draft, Validation, Methodology, Formal analysis, Data curation. GL: Writing – review & editing, Visualization, Validation. BH: Writing – review & editing, Visualization, Validation. CF: Writing – review & editing, Visualization, Validation, Supervision, Conceptualization.
